# Effects of Different Donor Ages on the Growth of Cutting Seedlings Propagated from Ancient *Platycladus orientalis*

**DOI:** 10.3390/plants12091754

**Published:** 2023-04-25

**Authors:** Yao Dong, Wei Guo, Wenfa Xiao, Jianfeng Liu, Zirui Jia, Xiulian Zhao, Zeping Jiang, Ermei Chang

**Affiliations:** 1 Key Laboratory of Forest Ecology and Environment of National Forestry and Grassland Administration, Ecology and Nature Conservation Institute, Chinese Academy of Forestry, Beijing 100091, China; 2 Taishan Academy of Forestry Sciences, Taian 271000, China; 3 State Key Laboratory of Tree Genetics and Breeding, Key Laboratory Tree Breeding and Cultivation of the National Forestry and Grassland Administration, Research Institute of Forestry, Chinese Academy of Forestry, Beijing 100091, China

**Keywords:** *Platycladus orientalis*, transcriptome, growth, cutting seedlings, ancient tree

## Abstract

The effects of tree age on the growth of cutting seedlings propagated from ancient trees have been an important issue in plant breeding and cultivation. In order to understand seedling growth and stress resistance stability, phenotypic measurements, physiological assays, and high-throughput transcriptome sequencing were performed on sown seedlings propagated from 5-year-old donors and cutting seedlings propagated from 5-, 300-, and 700-year-old *Platycladus orientalis* donors. In this study, the growth of cutting seedlings propagated from ancient trees was significantly slower; the soluble sugar and chlorophyll contents gradually decreased with the increase in the age of donors, and the flavonoid and total phenolic contents of sown seedlings were higher than those of cutting seedlings. Enrichment analysis of differential genes showed that plant hormone signal transduction, the plant–pathogen interaction, and the flavone and flavonol biosynthesis pathways were significantly up-regulated with the increasing age of cutting seedlings propagated from 300- and 700-year-old donors. A total of 104,764 differentially expressed genes were calculated using weighted gene co-expression network analysis, and 8 gene modules were obtained. Further, 10 hub genes in the blue module were identified, which revealed that the expression levels of *JAZ*, *FLS*, *RPM1/RPS3*, *CML*, and *RPS2* increased with the increase in tree age. The results demonstrated that the age of the donors seriously affected the growth of *P. orientalis* cutting seedlings and that cutting propagation can preserve the resistance of ancient trees. The results of this study provide important insights into the effects of age on asexually propagated seedlings, reveal potential molecular mechanisms, and contribute to an improvement in the level of breeding and conservation of ancient germplasm resources of *P. orientalis* trees.

## 1. Introduction

The conservation of germplasm resources of ancient and notable trees has been paid increasing attention [1]. Ancient *P. orientalis* has the characteristics of wide adaptability, strong biotic and abiotic resistance, great longevity, and so on [2]. However, due to climate and human factors, a large number of ancient *P. orientalis* trees have gradually withered, and it is particularly important to preserve the genetic materials. Cutting is now widely applied in the propagation of ancient tree species, and the age of the donors significantly affects the growth of the cutting seedlings [3]. Cutting seedlings of *Robinia pseudoacacia* and *Taxus globosa* propagated from young donors grew much better than those from mature trees [4,5]. There are differences between the seedlings propagated using different methods. For example, *hybrid poplar* seedlings propagated from tissue culture were superior to rooted cuttings for leaf area, leaf length, leaf width, and leaf dry mass [6]. Propagation methods also affect the anti-stress capability of trees. The rooted cuttings of *Passiflora edulis* were more tolerant to salt stress than sown seedlings and grafted seedlings. Thus, cutting seedlings are also better able to cope with environmental stresses and survive under extreme conditions [7]. However, the stability of the resistance phenotype of cutting seedlings from ancient trees has not been studied so far. Furthermore, the effects of donor age on the growth of cutting seedlings propagated from ancient trees should be studied in depth.

The age of donor trees is known to be related to the growth status and stress resistance of the cutting seedlings [8,9]. In previous studies, our group found that the peroxidase and superoxide dismutase activities were higher in ancient *P. orientalis* than those in 20-year-old trees, while the contents of soluble protein and chlorophyll were the opposite [10]. It has been demonstrated that the contents of photosynthetic pigments (chlorophyll a, chlorophyll b, and total chlorophyll) and soluble sugars decreased with age [11]. Under abiotic stresses, the over-expressed flavone metabolic and biosynthetic pathways can directly eliminate reactive oxygen species (ROS) in plants, thereby improving the drought tolerance of plants [12,13,14]. Phenolics play an essential role in the defense reactions of plants against pathogens, and the total phenolic content in plants is influenced by age [15,16], indicating that the accumulation of secondary metabolites such as flavonoids and total phenolics is significantly affected by plant age. Different tree ages can lead to different responses of plants to pathogens. For example, adult *Dianthus pavonius* is much more *resistant* to infection secondary to inoculation compared to the resistance of young [17]. Comparison experiments between hybrid poplar at different ages showed that young leaves are more susceptible to leaf spot infection than old leaves [18]. Whether the rooted seedlings can effectively preserve the stress resistance of ancient trees needs further research attention.

The regulatory mechanism for the growth and development of ancient trees may affect the growth of their cutting seedlings. For example, the expression patterns and function in some gene families may be affected by tree age, and these genes may be related to plant growth and development [19,20]. Expression of hormone-related genes, ROS scavenger genes, senescence-related genes, and defense response genes were up-regulated with tree age [21]. It has been reported that some genes belonging to the *AP2/ERF* and *bHLH* families affect plant growth and development by regulating the biosynthesis and signal transduction pathways of plant hormones. In addition, the expression of genes related to cell division, cell expansion, and differentiation was significantly reduced in ancient trees, especially *miR166* and *HD-ZIP III* involved in cambium activities. Disease resistance-associated genes and defensive secondary metabolites genes were found to be highly expressed in ancient trees [22]. However, the molecular mechanisms for differences in growth between seedlings propagated from donor trees at different ages have not been clarified.

With the advancement in forest tree breeding and development in forest industries, clonal forestry has played an increasingly important role in the cultivation of planted forests throughout the world. In recent years, with the popularization of molecular biology techniques, more and more studies have begun to focus on the mechanisms for the tree age effect on seedling growth. High-throughput sequencing techniques have made it possible to elucidate the effects of donor age on the growth of cutting seedlings. To make a comprehensive evaluation of the stability and reliability of seedlings propagated from ancient trees at different ages, phenotypic measurements, physiological assays, and high-throughput transcriptome sequencing were performed on sown seedlings propagated from 5-year-old donors and cutting seedlings propagated from 5-, 300-, and 700-year-old donors in this study. The aim of this study was to reveal key genes and metabolic pathways involved in the growth and development of *P. orientalis* seedlings with increasing donor age, which is of important guiding significance for the conservation and management of germplasm resources of excellent ancient trees using clonal propagation.

## 2. Results

### 2.1. Growth and Morphological Characteristics of Sown and Cutting Seedlings of P. orientalis Propagated from Donors at Different Ages

**Figure 1:**
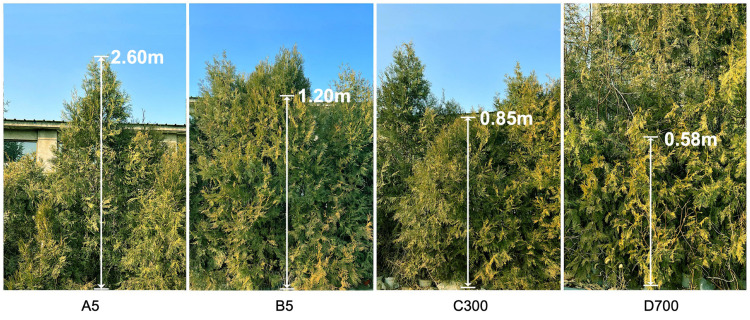
The 5-year-old sown seedlings and cutting seedlings propagated from 5-, 300-, and 700-year-old *P. orientalis* donors used as research material.

**Figure 2:**
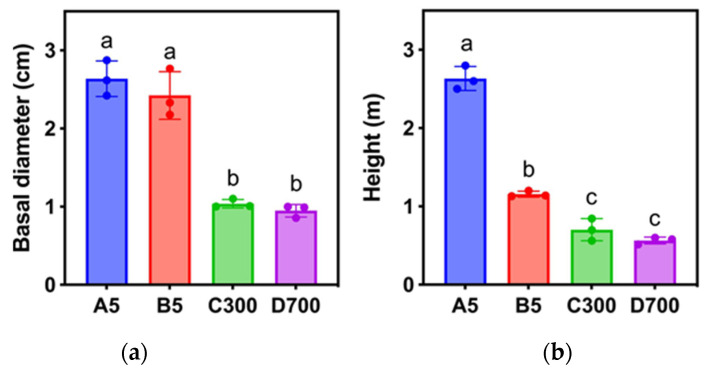
Variation in basal stem diameters and heights of sown seedlings and cutting seedlings of *P. orientalis* propagated from differently aged donors. (**a**) Basal stem diameters. (**b**) Heights. Note: A5: sown seedlings propagated from 5-year-old donors, B5: cutting seedlings propagated from 5-year-old donors, C300: cutting seedlings propagated from 300-year-old donors, and D700: cutting seedlings propagated from 700-year-old donors. Values are the mean of three replicates of samples, and bars represent standard errors. Means with the different letters are significantly different at *p* < 0.05 calculated using Duncan’s multiple range test.

The growth and morphological characteristics of cutting seedlings of *P. orientalis* propagated from donors at different ages were different (Figure 1). The results showed that the basal stem diameters and plant heights of cutting seedlings propagated from 5-year-old donors were significantly greater than those from 300- and 700-year-old donors (*p* < 0.05). No significant differences were found between sown and cutting seedlings, both of which were propagated from 5-year-old donors, and neither were there differences between cutting seedlings propagated from 300- and 700-year-old donors, respectively (Figure 2). Therefore, the age of donors significantly affected the growth of the rooted cuttings, and the growth of rooted cuttings propagated from ancient trees was significantly slower.

### 2.2. Analysis of Soluble Sugar, Chlorophyll, Flavonoid, and Total Phenol Content

**Figure 3:**
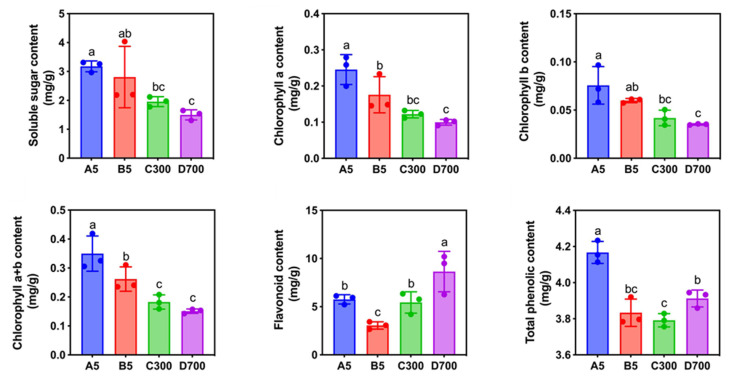
Changes in the contents of soluble sugar, chlorophyll a, chlorophyll b, chlorophyll a+b, flavonoid, and total phenol of sown and cutting seedlings propagated from differently aged donors of *P. orientalis*. Note: A5: sown seedlings propagated from 5-year-old donors, B5: cutting seedlings propagated from 5-year-old donors, C300: cutting seedlings propagated from 300-year-old donors, and D700: cutting seedlings propagated from 700-year-old donors. Values are the mean of three replicates of samples, and bars represent standard errors. Means with different letters are significantly different at *p* < 0.05 calculated using Duncan’s multiple range test.

The contents of soluble sugar and chlorophyll (Chl. a, Chl. b, and Chl. a + b) gradually decreased with the increase in the age of donors (Figure 3). The contents of Chl. a and Chl. (a + b) in sown seedlings propagated from 5-year-old donors were significantly greater than that of rooted cuttings propagated from 5-year-old donors. The contents of soluble sugar and chlorophyll in cutting seedlings propagated from 5-year-old donors were significantly greater than those from 300- and 700-year-old donors. This showed that the sown seedlings grew more vigorously than the cuttings. The contents of flavonoid and total phenol in sown seedlings propagated from 5-year-old donors were higher than those in cutting seedlings propagated from 5-year-old donors (Figure 3). The contents of flavonoid and total phenol in the cutting seedlings increased with the increasing age of donors.

### 2.3. Transcriptome Sequencing and Reassembly of P. orientalis Unigenes

A total of 105,530,209 reads were assembled into 104,764 unigenes, and the average length of the assembled unigenes was 1007.31 bp. The N50 value was 1858 bp, and the GC content was 38.75%. Assessment of the assembly integrity was performed using BUSCO scored C: 73.9%. There were 23,652 unigenes of 501–1000 bp, 9559 unigenes of 501–1000 bp, and 20,641 unigenes of >1500 bp. Most unigenes were in the range of 0–500 bp, with an abundance of 49% (Table 1), indicating that the sequencing data met the strict quality requirements for subsequent transcriptome analysis.

### 2.4. Functional Classification of P. orientalis Unigenes

To assign gene ontology annotation for the unigenes, the BLAST algorithm was used to search seven databases: NR, NT, Swiss-Prot, Pfam, COG, GO, and KEGG. The results showed 26,226 (54.58%) unigenes with homologous sequences in at least one database and 3105 (6.46%) unigenes in all seven databases. Among them, 24,080 unigenes (50.12% of all unigenes) were annotated with a significant BLAST result in the Nr database, 8380 unigenes (17.44%) were annotated in the Nt database, and 18,483 unigenes (38.47%) were annotated in the Swiss-Prot database. Annotation results for unigenes are shown in Table 2.

**Figure 4:**
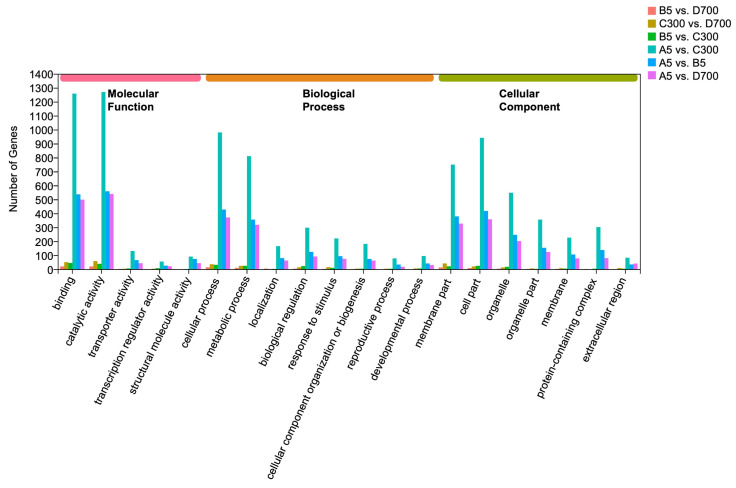
Gene ontology (GO) classifications of the assembled unigenes. The main functional annotations in the biological process, cellular component, and molecular function categories are presented. Bars represent the number of *P. orientalis* proteins with BLASTX matches to each GO term. Note: A5: sown seedlings propagated from 5-year-old donors, B5: cutting seedlings propagated from 5-year-old donors, C300: cutting seedlings propagated from 300-year-old donors, and D700: cutting seedlings propagated from 700-year-old donors.

In this study, a total of 104,764 unigenes were annotated in the GO database. The GO terms were placed in three categories including cellular component (6258 unigenes), biological process (5524 unigenes), and molecular function (5702 unigenes). The GO terms enriched in cellular components were cell part (1766 unigenes) and membrane part (1526 unigenes). The GO terms enriched in the biological process were cellular process and metabolic process, with 1856 and 1541 unigenes, respectively. There were only 109 and 207 unigenes in molecular function with transcription regulator activity and structural molecule activity, respectively (Figure 4).

**Figure 5:**
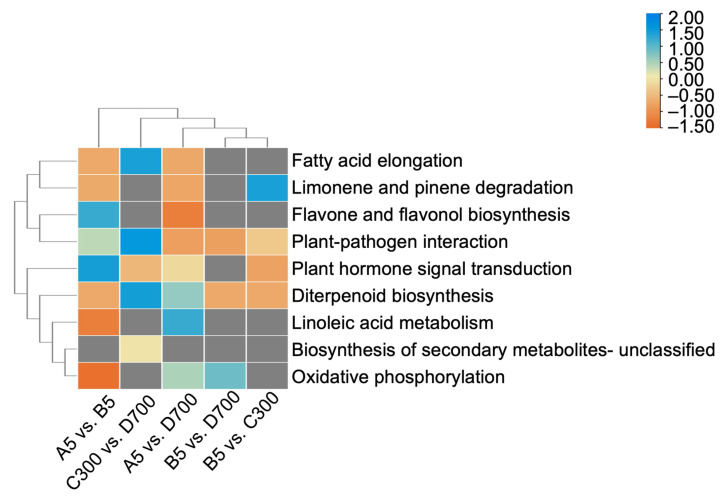
Heatmap showing unigenes based on the Kyoto Encyclopedia of Genes and Genomes (KEGG) categorizations in pairwise comparisons. Significantly enriched KEGG pathways (corrected *p*-value < 0.05) in five comparisons (A5-/B5-year-old, C300-/D700-year-old, A5-/D700-year-old, B5-/D700-year-old, and B5/C300-year-old). Note: A5: sown seedlings propagated from 5-year-old donors, B5: cutting seedlings propagated from 5-year-old donors, C300: cutting seedlings propagated from 300-year-old donors, and D700: cutting seedlings propagated from 700-year-old donors.

Unigenes were mainly enriched in nine KEGG pathways. The results indicate that the plant hormone signal transduction pathway has the most differentially expressed genes (DEGs) in B5 vs. C300, while the DEGs in A5 vs. B5 and A5 vs. D700 were mostly enriched in the flavone and flavonol biosynthesis pathways. In addition, the plant–pathogen interaction pathway was significantly enriched with DEGs in A5 vs. D700 (Table 3, Figure 5). Therefore, gene expression may be affected by the propagation method and age of the donor. With increasing tree age, the cuttings of *P. orientalis* had greater stress resistance and hormone signal transduction capabilities. The biosynthesis of flavone and flavonol in cutting seedlings propagated from 700-year-old donors was enhanced, and secondary metabolism was improved in cutting seedlings propagated from 5-year-old donors, suggesting that they grew vigorously, while the cutting seedlings propagated from 300- and 700-year-old donors had a higher degree of lignification.

### 2.5. Functional Classification of DEGs

**Figure 6:**
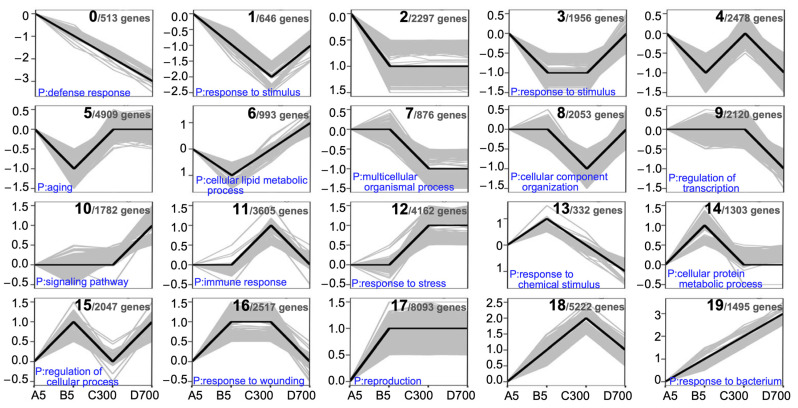
K-means clustering of gene expression profiles. Genes with different expression patterns were grouped into 20 clusters. The number of genes in each cluster and the functional classification of each cluster are shown. Note: A5: sown seedlings propagated from 5-year-old donors, B5: cutting seedlings propagated from 5-year-old donors, C300: cutting seedlings propagated from 300-year-old donors, and D700: cutting seedlings propagated from 700-year-old donors.

To further investigate the functional classifications of DEGs, we performed a K-means cluster analysis, and the 104,764 unigenes were classified into 20 functional cluster profiles (Figure 6). The genes in profile 19 were mainly involved in the response to bacterium. These genes, which had higher expression levels in cutting seedlings than in sown seedlings, were continuously up-regulated with increasing age, indicating that the cutting seedlings of ancient trees have greater resistance to pathogens. Profile 3 and profile 10 were mainly enriched in response to stimulus and signaling pathways, and they were up-regulated in the cutting seedlings propagated from 700-year-old donors, suggesting that with the influence of donor age, the cutting seedlings propagated from 700-year-old donors grew with modulation of complex defensive response. The genes involved in profile 17, enriched in reproduction, had higher expression levels in sown seedlings than in cutting seedlings, both of which were propagated from 5-year-old donors, and then their expression levels tended to be stable.

Genes involved in profile 0 were enriched in the defense response and were continuously down-regulated with increasing tree age, indicating that the resistance ability of these cutting seedlings was affected by the age of the donors. Genes involved in profiles 1 and 18 exhibited a trend in decreased expression levels except for seedlings propagated from 300-year-old donors. GO functional enrichment demonstrated that these genes were enriched in response to stimulus and cellular component organization. In the three groups of cutting seedlings propagated from donors at different ages, expression levels of the genes in profile 2 were stable, suggesting that the expression of these genes was related to the propagation method.

### 2.6. Changes in Age-Related Changes in Three Enrichment Pathways

**Figure 7:**
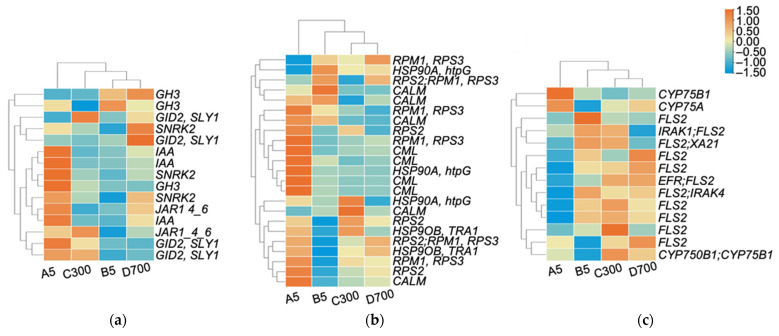
Genes representing different functional classes and showing age-dependent changes in transcript levels: (**a**) plant hormone signal transduction, (**b**) plant–pathogen interaction, and (**c**) flavone and flavonol biosynthesis. Note: A5: sown seedlings propagated from 5-year-old donors, B5: cutting seedlings propagated from 5-year-old donors, C300: cutting seedlings propagated from 300-year-old donors, and D700: cutting seedlings propagated from 700-year-old donors.

The enriched KEGG pathways included plant hormone signal transduction, the plant–pathogen interaction, and flavone and flavonol biosynthesis, the genes of which were significantly up-regulated in cutting seedlings propagated from ancient donors (Figure 7). *GID2/SLY1* (Gibberellin-insensitive dwarf 2/Slender Rice 1), *JAR1* (Jasmonate Resistant 1), *EFR* (Elongation factor Tu receptor), *FLS2* (Flavonol synthase 2) and *CYP75A* (Flavonoid 3′,5′-hydroxylase) were up-regulated with increasing tree age, indicating that with the increase in tree age, cutting seedlings have stronger resistance to stresses. The expression levels of genes simultaneously involved in plant hormone signal transduction and the plant–pathogen interaction in sown seedlings propagated from 5-year-old donors were higher than those in cutting seedlings propagated from 5-, 300-, and 700-year-old donors. These pathways included *SNRK2* (Sucrose non-fermenting-1-related protein kinase 2s) and *JAR1_4_6*, as well as the anti-disease genes *RPM1/RPS3* (Resistance to *Pseudomonas syringae* pv. *maculicola* 1), *RPS2* (Resistance to *P. syringae* 2) and *CML* (Calmodulin-like protein)*,* suggesting that cutting seedlings were less resistant to stress than sown seedlings.

### 2.7. Weighted Gene Co-Expression Network Analysis (WGCNA) Identifying Conserved Differentially Expressed Genes

**Figure 8:**
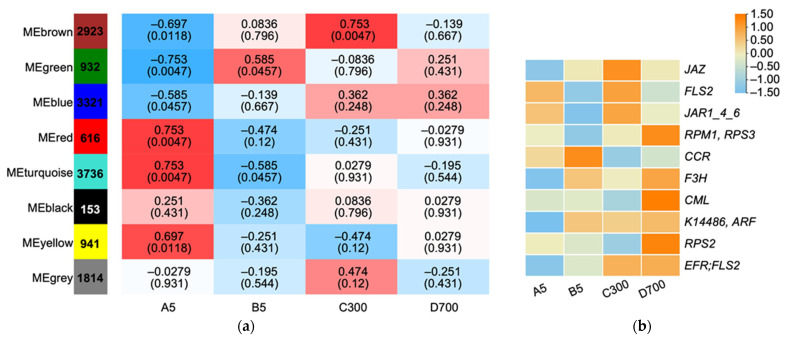
(**a**) Correlations between modules and traits. The horizontal axis represents different samples, and the vertical axis represents different modules. The column of numbers on the left represents the number of genes in the module, and each group of data on the right represents the correlation coefficients and significant *p*-values between the modules and the phenotypes. Red and blue represent large and small correlations between modules and phenotypes, respectively. (**b**) Genes representing different functional classes and showing age-dependent changes in transcript levels in blue modules. Note: A5: sown seedlings propagated from 5-year-old donors, B5: cutting seedlings propagated from 5-year-old donors, C300: cutting seedlings propagated from 300-year-old donors, and D700: cutting seedlings propagated from 700-year-old donors.

The 104,764 DEGs were calculated using WGCNA, and 8 modules were obtained. The trend in gene expression levels in the blue module was significantly correlated with increasing tree age (*R* = −0.585, *P* = 0.0457; Figure 8a), suggesting that genes in this module were positively associated with age in *P. orientalis*. The blue module contained 3321 DEGs, most of which were highly expressed in seedlings propagated from 300- and 700-year-old donors. A total of 17 genes related to the plant–pathogen interaction were involved in the MAPK signaling pathway, including 7 members of the plant receptor-like kinase *FLS2* gene family. *FLS2* is involved in recognizing plant flagellin as a signal of bacterial existence and activating the defense response [23], and its expression levels gradually increased during the growth of *Arabidopsis thaliana* seedlings [24]. In the blue module, ten genes considered hub genes were involved in key biosynthetic pathways. The expression levels of four plant–pathogen interaction-related genes (*EFR/FLS2*, *RPM1/RPS3*, *CML*, and *RPS2*) in the module increased with increasing tree age (Figure 8b), indicating that cutting seedlings retain the strong stress resistance of ancient trees. These results indicated that the pathogen defense capability of *P. orientalis* cutting seedlings was gradually enhanced with the increasing age of the donors.

### 2.8. Identification of Hub Genes in the Blue Module

Ten genes in the blue module were involved in key biosynthetic pathways, and they were considered hub genes in the module. The expression levels of four plant–pathogen interaction-related genes (*EFR/FLS2*, *RPM1/RPS3*, *CML*, and *RPS2*) in the module increased with the increase in tree age (Figure 8b), indicating that cutting seedlings retain the strong stress resistance of ancient trees. Among the two genes (*CCR* (Cinnamoyl-CoA reductase) and *F3H* (Flavanone 3-hydroxylase)) involved in flavonoid biosynthesis, the expression trend in *F3H* increased with tree age. *F3H* plays an important role in plant fiber development and the anthocyanin synthesis pathway [25]. The differential expressions of these genes affect the accumulation of secondary metabolites and leaf color in cutting seedlings. *JAZ* (Jasmonate ZIM-domain protein) and *JAR1_4_6*, and *K14486/ARF* (Auxin response factor) participate in plant hormone signal transduction, among which *JAZ* and *JAR1_4_6* are members of the *JAZ* family and are highly expressed in ancient tree cutting seedlings. *JAZ* is a key regulator of the JA signaling pathway, which can regulate the biosynthesis of secondary metabolites such as terpenes, alkaloids, and flavonoids. This suggests that with the increase in the age of cutting seedlings, their secondary metabolism increases, and they become more sensitive to stress.

### 2.9. qRT-PCR Validation of Selected Genes from Blue Module

**Figure 9:**
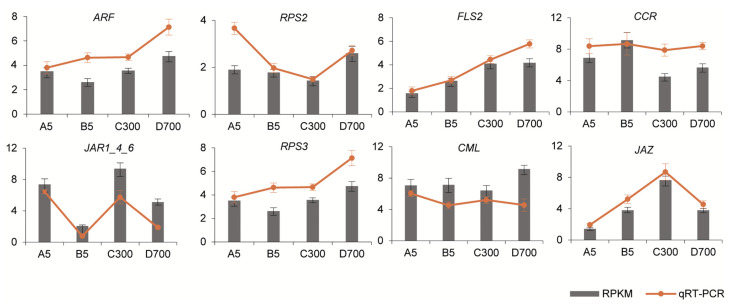
Comparison of the expression profiles for eight representative genes measured using RNA-seq and qRT-PCR. Columns represent expression levels determined using RNA-seq in FPKM values, while lines represent expression levels determined using qRT-PCR. Note: A5: sown seedlings propagated from 5-year-old donors, B5: cutting seedlings propagated from 5-year-old donors, C300: cutting seedlings propagated from 300-year-old donors, and D700: cutting seedlings propagated from 700-year-old donors. The error bars represent the SD from three replicates. The differential expression analysis was conducted using the 2^−ΔΔct^ method.

The expression levels of eight hub genes associated with plant hormone signal transduction, the plant–pathogen interaction, and phenylpropanoid biosynthesis in the blue module in the WGCNA analysis were determined using qRT-PCR. The primers for the eight selected unigenes are listed in Supplementary Table S1. The expression patterns in the eight candidate unigenes were similar to those in the transcriptome data (Figure 9). Therefore, the qRT-PCR analysis verified the data from the RNA-seq analysis, showing that our results are high reliability.

## 3. Discussion

### 3.1. Increasing Age of the Donors Affected the Basal Stem Diameter and Plant Height of Cutting Seedlings

Cuttage propagation has a positive significance for preserving excellent germplasm resources with stress resistance from ancient *P. orientalis*, and tree age significantly affects the growth and stress resistance of cutting seedlings [26]. In this study, the basal stem diameter and plant height of cutting seedlings decreased significantly with the increase in the age of donors, and sown seedlings grew more vigorously than cutting seedlings, both of which were propagated from 5-year-old donors. This is consistent with the research results reported indicating that the height, diameter, and rooting ability of *Larix laricina* decreased with aging [27]. The height of *Picea abies* cutting seedlings was significantly greater than that of sown seedlings [28]. Changes in the contents of soluble sugars and chlorophyll can be used as important physiological indicators for evaluating plant growth. The soluble sugar and chlorophyll contents of *Populus tremuloides* decreased with aging, respectively [29]. The soluble sugar and chlorophyll contents in cutting seedlings propagated from 5-year-old *P. orientalis* donors were greater than those from 300- and 700-year-old trees. Collectively, donor age has effects on the growth of cutting seedlings, and there is a decreasing tendency as the age of the tree increases. This may be due to the fact that ancient trees have more complex branching systems and higher requirements for environmental conditions, such as temperature, water, and nutrients, and are more susceptible to external environmental influences [30,31], which may lead to limitations in the growth of cutting seedlings propagated from ancient trees as well.

### 3.2. Increasing Age of the Donors Affected the Changes in Physiological Indexes of Cutting Seedlings

Flavonols participating in cell wall hardening are important in the plant stress response [32]. Flavonoid and flavonol biosynthetic pathway-related genes are up-regulated in old *Ginkgo Biloba* leaves [22], which is consistent with the up-regulation of *FLS2* and *CYP75A* in 300- and 700-year-old *P. orientalis* seedlings in this study. The transcription levels of the *FLS2* and *CYP75* family genes increased with increasing tree age. Research on *Taxus chinensis* came to a similar conclusion [33]. *CYP75* belongs to the largest cytochrome *P450* (*CYP*) superfamily of the plant enzyme family for plant metabolism, which participates in the biosynthesis of most plant secondary metabolites to adapt to biotic and abiotic stresses [34,35]. Both *CCR* and *F3H* are involved in flavonoid biosynthesis, and the expression level of *F3H* in *P. orientalis* cutting seedlings increased with donor age. *F3H* plays an important role in plant fiber development and the anthocyanin synthesis pathway [25]. Secondary metabolism in plants gradually increases with the age of the tree, which may be due to the fact that old seedlings are affected by various internal and external factors during their long-term growth and gradually develop their own defense mechanisms to cope with environmental challenges, and these defense mechanisms can be realized through the synthesis of secondary metabolites [36,37]. The secondary metabolite content and related gene expression in cutting seedlings are influenced by the donor trees. Thus, the age of the donors also has effects on the expression of secondary metabolite-related genes in *P. orientalis* cutting seedlings.

### 3.3. Regulation of Hub Gene in the Stress Resistance of Ancient Cutting Seedlings

Trees at different ages respond differently to pathogens. The age of the donor trees affects the function of the immune system in the cutting seedlings and the functioning of the resistance mechanisms to pathogens. This study showed that plant–pathogen interaction pathway-related disease-resistance proteins *RPM1/RPS3* and *CML* were up-regulated in cutting seedlings propagated from 300- and 700-year-old donors. *RPS3* can endow *P. syringae* strains with specific disease resistance [38]. *CML* is a calmodulin-like protein that promotes plant innate immunity through a flagellin-dependent signaling pathway [39]. *CML* in *P. abies* promotes the root growth of seedlings by sensing the fluctuation in cytoplasmic Ca^2+^ during the stress response [40]. The regulation of these genes involved in stress resistance in cutting seedlings propagated from ancient *P. orientalis* needs further study in the future. Seedlings propagated from cuttings are genetically identical to the donor trees, and their genetic background is similar to that of the donor trees, so their disease resistance may also be similar to that of the donor tree [41]. These results indicate that cuttage can retain the growth properties of ancient *P. orientalis*, especially stress resistance.

GA regulates plant growth and defense in response to various stimuli [42]. Our data showed that GA and JA synthesis-related genes such as *GID2/SLY1* and *JAR1* were up-regulated in cutting seedlings propagated from 300- and 700-year-old *P. orientalis* donors. *GID2*, a receptor similar to hormone-sensitive lipase in gibberellin, plays a role in the response to stresses [43,44]. *JAR1* up-regulation was consistent with the research results indicating that JA-related genes were up-regulated in aged *A. thaliana* [45]. However, the expression levels of genes involved in the JA synthesis pathway decreased in aged *G. biloba* branches [46]. Therefore, the regulatory roles of JA in cutting seedlings propagated from 300- and 700-year-old ancient *P. orientalis* need further study. These results demonstrate that cutting propagation well preserves the ability of aged trees to enhance their resistance to biotic and abiotic stresses, as well as excellent genetic resources.

**Figure 10:**
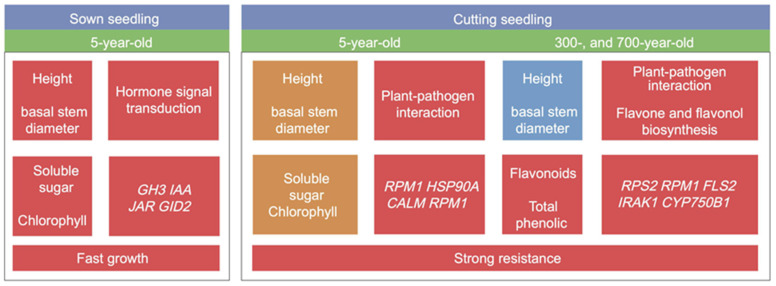
Framework plots showing phenotypes, physiological indicators, and molecular expression levels of sown seedlings propagated from 5-year-old donors and cutting seedlings propagated from 5-, 300-, and 700-year-old donors. Red represents high values, yellow represents medium values, and blue represents low values.

Based on the collective results of this study, we constructed framework plots showing phenotypes, physiological indicators, and molecular expression levels of sown seedlings propagated from 5-year-old donors and cutting seedlings propagated from 5-, 300-, and 700-year-old donors (Figure 10).

## 4. Materials and Methods

### 4.1. Plant Materials and Growth Conditions

At a point in the growing season (June 2021), healthy growing sown seedlings propagated from 5-year-old *P. orientalis* donors were selected as controls from the experimental nursery of the Chinese Academy of Forestry located in Beijing. The experimental materials were 5-year-old *P. orientalis* cutting seedlings propagated from donors at different ages (5-, 300-, and 700-year-old). Phenotype characterization, physiological tests, and transcriptomic analysis were carried out with three biological replicates per experiment. The freshly collected leaf material was immediately frozen in liquid nitrogen and stored at −80 °C until processing for total RNA extraction.

### 4.2. Phenotypic Measurements

A completely randomized design was performed throughout the experiments. Basal stem diameters of seedlings were measured at 2 cm above the ground using an INSIZE 1108-150 electronic caliper, and the heights were measured from the base of the main stem to the tip of the highest leaf with a tape measure.

### 4.3. Determination of Soluble Sugar and Chlorophyll Contents

Approximately 0.2 g of healthy seedling leaves were harvested for the measurement of the soluble sugar content. Soluble sugar was analyzed using an anthrone colorimetric method [47].

Chlorophyll was extracted with approximately 0.1 g ground leaf material using acetone and 90% ethanol (*v*/*v*) at 4 °C under low light intensity for 3 h until the leaf material turned blanch. Chlorophyll a, chlorophyll b, and total chlorophyll contents were determined using absorbance readings.

### 4.4. Determination of Flavonoids and Total Phenolics

Total flavonoid content of the prepared extracts was determined using the aluminum chloride method [48]. First, 0.02 g of finely ground leaf material was extracted with 60% ethanol (*v*/*v*) at 60 °C. Then, 108 µL of supernatant was taken from the top of the tube, and 6 µL of NaNO_2_ (1:20 *w*/*v*) and 6 µL of AlCl_3_ (1:10 *w*/*v*) was added and then incubated for 6 min at 25 °C. Then, 80 µL sodium hydroxide (1 N) was added to the mixture. Finally, after mixing thoroughly, the absorbance was measured at 510 nm with a UV visible spectrophotometer using distilled water as the blank. The standard calibration curve was prepared using Rutin ranging from 0 to 1200 µg/mL. The content of flavonoids was determined using the absorbance.

The total phenolic content was determined using the Folin–Ciocalteu colorimetric method [49]. Briefly, 0.1 g of finely ground leaf material was extracted with 60% ethanol (*v*/*v*) for 2 h with shaking at 60 °C. Then, 10 µL of the extracted sample was mixed with 50 µL of Folin–Ciocalteu reagent at room temperature for 2 min. Next, 50 µL of 5% (*w*/*v*) sodium carbonate solution was added to stop the reaction, and then 90 µL of distilled water was added to make 200 µL. The mixture was incubated at 25 °C for 10 min, and the absorbance was measured at 760 nm. Gallic acid content ranging from 0 to 300 µg/mL was prepared, and the calibration curve was obtained using a linear fit (r^2^ = 0.9961).

### 4.5. Transcriptome Sequencing Methods

Total RNA was extracted using an RNAprep Pure Plant Kit (Tiangen, Beijing, China). The quality of the RNA was assessed using gel electrophoresis, and the concentration was measured using a NanoDrop 2000 spectrophotometer (Thermo Scientific, Wilmington, DE, USA). Then, cDNA libraries were constructed with RNA and sequenced using the Illumina NovaSeq 6000 platform. Raw reads were cleaned by removing adaptors and low-quality sequences. Clean reads were then aligned to the reference genome using the spliced read mapper TopHat2 (version 2.0.7) [50] and also mapped to the de novo *P. orientalis* transcriptome assembly using Cufflinks (version 2.2.1) [51]. Protein-coding genes were predicted using the NCBI NR, SWISS-PROT, GO, COG, KOG, eggNOG, and KEGG databases.

### 4.6. Quantitative Real-Time PCR Analysis

First-strand cDNA was synthesized from 1 µg total RNA using a Prime Script™ Reagent Kit (Takara, Dalian, China). The quantitative real-time PCR was performed using KAPA SYBR FAST qPCR Master Mix (Kapa Biosystems, Boston, MA, USA). Primers were designed using Premier-BLAST [52], and the sequences are depicted in Supplementary Table S1. Thermal cycling conditions were as follows: 5 s at 95 °C and 30 s at 60 °C for 40 cycles. *Actin* was selected as a reference gene [53], and the relative expression data were calculated using the 2^−ΔΔct^ method [54].

### 4.7. Statistical Analysis of Data

The significance of differences among the mean values for growth and physiological characteristics were calculated using one-way analysis of variance (ANOVA) and expressed as the mean ± standard error (SE), followed with a Duncan’s test at *p* < 0.05. All statistical analyses were performed with SPSS 22.0 (SPSS Inc., Chicago, IL, USA). Transcript levels were quantified as fragments per kilobase of transcript per million mapped reads (FPKM). DEGs were identified using the *R* package DESeq2 (V 1.32.0) [55]. Important DEGs were identified according to the following criteria: each paired comparison |fold-change| ≥ 2 and FDR ≤ 0.001. The number of DEGs in different comparisons was visualized using the *R* package UpSetR (version 1.4.0) [56]. The weighted gene co-expression network was constructed using the WGCNA package in *R* software. Soft thresholds were calculated using the pick soft threshold function provided by WGCNA, and the gene cluster heat map was drawn using Tbtools [57].

## 5. Conclusions

This study combined growth, physiological indicators, and transcriptome sequencing to comprehensively analyze the effects of *P. orientalis* donors at different ages on the growth of cutting seedlings. Donor age affected the basal stem diameter and plant height of cutting seedlings, and the sown seedlings grew markedly better than the cutting seedlings. With increasing donor age, the contents of soluble sugars and chlorophyll decreased, while the content of secondary metabolites increased, and the expression levels of plant hormone signal transduction, the plant–pathogen interaction, and flavone and flavonol biosynthesis pathway-related genes (*JAZ*, *FLS*, *RPM1/RPS3*, *CML*, and *RPS2*) were significantly up-regulated. The results demonstrated that the growth and stress resistance of *P. orientalis* cutting seedlings were greatly affected by the age of donors and that cutting seedlings can maintain the stress resistance of ancient trees, also indicating the reliability and stability of clonal propagation. These results provide a theoretical basis for revealing the molecular mechanisms underlying the effects of donor age on the growth of cutting seedlings and are important guidelines for the conservation and management of excellent ancient tree germplasm resources using asexual propagation. Future research needs to combine the photosynthetic characteristics of seedlings, other physiological indicators, and multi-omics in a comprehensive analysis to investigate the effects of ancient tree age on cutting seedlings in depth.

## Figures and Tables

**Table 1:** De novo assembly of the *P. orientalis* transcriptome.

Items	Number
Total number	104,764
Total base	105,530,209
Average length (bp)	1007.31
N50	1858
GC percent	38.75%
BUSCO score	C: 73.9%
Number of transcripts between 0 and 500	50,912 (49%)
Number of transcripts between 501 and 1000	23,652 (23%)
Number of transcripts between 1001 and 1500	9559 (9%)
Number of transcripts >1500	20,641 (19%)

**Table 2:** Functional annotation of the *P. orientalis* transcriptome.

	Number of Unigenes	Percentage (%)
Annotated in NR	24,080	50.12
Annotated in NT	8380	17.44
Annotated in KO	7783	16.19
Annotated in SwissProt	18,483	38.47
Annotated in PFAM	18,546	38.6
Annotated in GO	20,029	41.68
Annotated in KOG	9877	20.55
Annotated in all Databases	3105	6.46
Annotated in at least one Database	26,226	54.58
Total unigenes	48,044	100

**Table 3:** Number of DEGs in pairwise comparisons.

	Specific Up-Regulated	Specific Down-Regulated	PPI (Nodes)	Percentage
A5 vs. B5	1225	384	73	21.60%
A5 vs. C300	3319	683	123	53.80%
A5 vs. D700	932	556	45	20%
B5 vs. C300	55	69	73	1.70%
B5 vs. D700	27	18	14	0.60%
C300 vs. D700	50	121	8	2.30%

## Data Availability

The raw sequencing data generated from this study have been deposited in NCBI SRA (https://www.ncbi.nlm.nih.gov/sra, accessed on 15 April 2023) under the accession number PRJNA947522.

## Supplementary Materials

The following supporting information can be downloaded at: https://www.mdpi.com/article/10.3390/plants12091754/s1, Table S1: Primers for the candidate genes designed for qRT-PCR.
