# Supplementary material for: Effects of Different Donor Ages on the Growth of Cutting Seedlings Propagated from Ancient Platycladus orientalis
Source: Plants (Basel). 2023 Apr 25;12(9):1754. doi: 10.3390/plants12091754 (PMC10181453; doi:10.3390/plants12091754)
Supplement: Supplementary file 1 [file plants-12-01754-s001.zip › plants-2328756-supplementary.pdf]

Supplementary Table S1. Primers of the candidate genes designed for qRT-PCR.

Gene ID	Gene abbreviation	Gene name	Size (bp)	Forward primer<br>Reverse primer
TRINITY_DN73607_c0_g1	*ARF*	*Auxin response factor 4*	113	TGTTCGACAAGTGCAAGAGG<br>CACCTTGTCTCCTCCCAAAA
TRINITY_DN7525_c0_g2	*RPS2*	*Resistance to Pseudomonas syringae 2*	140	CTTGGAAGGTTTGTGGAGGA<br>CATGGTGGCAGTTTTGTCTG
TRINITY_DN984_c0_g3	*FLS2*	*Elongation factor Tu receptor*	95	TCCCATACAATCCCACCAAG<br>CGCCTAGCTCAATGGTTGAT
TRINITY_DN3540_c0_g2	*CCR*	*Cinnamoyl-CoA reductase*	123	TATCGAACCGGAGCAGAATC<br>TGCGAGCTAAGAAAGGGAAA
TRINITY_DN27271_c0_g2	*JAR1_4_6*	*Jasmonate Resistant 1*	136	GAGGTGCTTGGAGTCCTGAA<br>TTTCCACATCGAAGGAGACC
TRINITY_DN2934_c0_g1	*RPS3*	*Resistance to Pseudomonas syringae pv. maculicola 1*	80	GCGGTATGGGTTGTAGTGCT<br>GTCGTTGCCGAAGTACCAAT
TRINITY_DN6298_c0_g1	*CML*	*Calmodulin-like protein*	81	GCGCAAGTTTGGCTCTTTAC<br>AGCCCATGAATCATTTGAGC
TRINITY_DN12112_c0_g1	*JAZ*	*Jasmonate ZIM-domain protein*	93	CCAAAGGGCCATTTTGTTGT<br>CGTGGTTGAAGCCAAATTCT
